# A Critical Appraisal of Strategies to Optimize Vitamin D Status in Germany, a Population with a Western Diet

**DOI:** 10.3390/nu11112682

**Published:** 2019-11-06

**Authors:** Roman Saternus, Thomas Vogt, Jörg Reichrath

**Affiliations:** 1 Center for Clinical and Experimental Photodermatology, Saarland University, Campus Homburg, 66421 Homburg, Germany; Thomas.vogt@uks.eu (T.V.); Joerg.Reichrath@uks.eu (J.R.); 2 Department of Dermatology, The Saarland University Hospital, 66421 Homburg, Germany

**Keywords:** vitamin D status, Germany, Dietary Intake of vitamin D, UVB-induced cutaneous vitamin D production

## Abstract

During the last decade, our scientific knowledge of the pleiotropic biological effects of vitamin D metabolites and their relevance to human health has expanded widely. Beyond the well-known key role of vitamin D in calcium homeostasis and bone health, it has been shown that vitamin D deficiency is associated with a broad variety of independent diseases, including several types of cancer, and with increased overall mortality. Moreover, recent findings have demonstrated biological effects of the vitamin D endocrine system that are not mediated via activation of the classical nuclear vitamin D receptor (VDR) by binding with high affinity to its corresponding ligand, the biologically active vitamin D metabolite 1,25-dihydroxyvitamin D (1,25(OH)_2_D). In contrast, many of these new biological effects of vitamin D compounds, including regulation of the circadian clock and many metabolic functions, are mediated by other vitamin D metabolites, including 20-hydroxyvitamin D and 20,23-dihydroxyvitamin D, and involve their binding to the aryl hydrocarbon receptor (AhR) and retinoid-orphan receptor (ROR). In most populations, including the German population, UVB-induced cutaneous vitamin D production is the main source for fulfilling the human body’s requirements of vitamin D. However, this causes a dilemma because solar or artificial UVR exposure is associated with skin cancer risk. In addition to UVB-induced vitamin D production in skin, in humans, there are two other possible sources of vitamin D: from diet and supplements. However, only a few natural foods contain substantial amounts of vitamin D, and in most populations, the dietary source of vitamin D cannot fulfill the body´s requirements. Because an increasing body of evidence has convincingly demonstrated that vitamin D deficiency is very common worldwide, it is the aim of this paper to (i) give an update of the vitamin D status in a population with a western diet, namely, the German population, and to (ii) develop strategies to optimize the vitamin D supply that consider both the advantages as well as the disadvantages/risks of different approaches, including increasing vitamin D status by dietary intake, by supplements, or by UVB-induced cutaneous synthesis of vitamin D.

## 1. Introduction

Due to the ubiquitous availability of solar ultraviolet radiation (UVR, includes UVA, UVB and UVC) in most regions worldwide, especially during spring and summer, and because most foods contain little vitamin D, cutaneous vitamin D production represents the most important source of vitamin D for humans [1,2,3,4].

During the last decade, our scientific knowledge of the pleiotropic biological effects of vitamin D metabolites and their relevance to human health has expanded widely. Beyond the well-known key role of vitamin D in calcium homeostasis and bone health, it has been shown that vitamin D deficiency is associated with a broad variety of independent diseases, including several types of cancer, cardio-vascular, autoimmune and infectious diseases [5]. Notably, the causal relationship between vitamin D status and health has convincingly been demonstrated for many outcomes, including overall mortality. As an example, in a systematic review that updated and reassessed the benefits and harms of vitamin D supplementation used in primary and secondary prophylaxis of mortality, vitamin D supplementation decreased mortality in all 56 trials analyzed together (5,920/47,472 (12.5%) vs. 6,077/47,814 (12.7%); risk ratio (RR) 0.97 (95% confidence interval (confidence interval (CI))) 0.94 to 0.99); *p* = 0.02; I(2) = 0%). ‘Worst-best case’ and ‘best-worst case’ scenario analyses demonstrated that vitamin D could be associated with a dramatic increase or decrease in mortality [6]. Two recent randomized controlled trials reported in the secondary analyses that cancer and progression to diabetes were reduced for those with BMI <25 and <30, respectively (for 2000 and 4000 IU per day vitamin D_3_ intake, respectively (1 IU is equivalent to 0.025 µg)) [7,8].

Moreover, recent findings have demonstrated biological effects of the vitamin D endocrine system (VDES) that are not mediated via activation of the classical nuclear vitamin D receptor (VDR) by binding with high affinity to its corresponding ligand, the biologically active vitamin D metabolite 1,25-dihydroxyvitamin D (1,25(OH)_2_D). In contrast, many of these new biological effects of vitamin D compounds, including regulation of the circadian clock and many metabolic functions, are mediated by other vitamin D metabolites, including 20-hydroxyvitamin D and 20,23-dihydroxyvitamin D, and involve their binding to and activation of the aryl hydrocarbon receptor (AhR) and/or retinoid-orphan receptor (ROR) [9,10,11].

It should be noted that the human body’s requirements for vitamin D can be fulfilled by at least three different approaches: the UVB-induced cutaneous synthesis of vitamin D [3] and the uptake of vitamin D from the diet and/or by supplements [12]. However, only a few foods naturally contain vitamin D in substantial amounts, and in most populations, food fortification and/or supplements are not sufficiently used. As a consequence, in most populations, the human body’s requirements for vitamin D must be fulfilled physiologically mainly by the UVB-induced cutaneous synthesis of vitamin D [3]. This causes a dilemma because exposure of the skin to solar or artificial UVR is associated with increased skin cancer risk [13]. 

Because of the high prevalence of vitamin D deficiency worldwide and because of the substantial consequences for human health, it is of high importance to obtain reliable data concerning vitamin D status and vitamin D supply in individual populations [14,15]. Vitamin D deficiency has been previously defined as a 25(OH)D serum concentration < 50 nmol/L (<20 ng/mL) and vitamin D insufficiency as a 25(OH)D serum concentration of 51–74 nmol/L (21–29 ng/mL) [16]. German authorities also consider a value below 50 nmol/L to be insufficient [17]. Some authors recommend a 25(OH)D serum concentration above ≥ 50 nmol/L (20 ng/mL) as being optimal for health [15]. The Endocrine Society clinical practice guideline recommends for children and adults who are vitamin D deficient a target value of 25(OH)D above 75 nmol/L (30 ng/mL) [18]. However, while there is general consensus that 25(OH)D serum concentrations below 25–30 nmol/L (10–12 ng/mL) need to be elevated to prevent and/or treat rickets and osteomalacia, there is currently no general consensus both on the optimal vitamin D serum level and in which (risk) groups of people the 25(OH)D serum concentration should be measured at all [15].

On the other hand, 25(OH)D concentrations higher than 375 nmol/L (150 ng/mL) have been defined to be the hallmark of vitamin D toxicity due to vitamin D overdosing [19]. 

Some papers reported U-shaped 25(OH)D concentration–health outcomes [20,21,22]. For example, in an analysis by Melamed et al. (13,331 adults ≥20 years), the authors found an increased rate of mortality in females with 25(OH)D levels >125 nmol/l (>50 ng/mL) [21]. However, the significance of the reported U-shaped associations is highly controversial [22]. Grant et al. discussed in a recent review article that a highly plausible reason for these findings could be that in many individuals with relatively high 25(OH)D serum concentration, their high 25(OH)D concentration is caused because they are at present treated for vitamin D deficiency or insufficiency. Their increased risk for health disorders and diseases may not be the result of their current relatively high vitamin D status but due to the previous long-lasting vitamin D deficiency or insufficiency [22]. Furthermore, recent investigations demonstrated that relative high levels of 25(OH)D serum concentration are associated with a greater benefit for several health outcomes, for example decreased risk in breast cancer (25(OH)D concentration ≥150 nmol/L compared with <50 nmol/L (hazard ratio (HR) = 0.20, *p* = 0.03)) [23], substantial reduction in preterm birth risk (maternal 25(OH)D concentration ≥ 100 nmol/L compared with <50 nmol/L (OR = 0.41, *p* = 0.002)) [24], substantial reduction in risk of all invasive cancers combined (25(OH)D concentration ≥100 nmol/L compared with <50 nmol/L (HR = 0.33, 95% CI = 0.12–0.90)) [25], improved control of systolic and diastolic blood pressure (BP) in hypertensive individuals who were vitamin D insufficient (25(OH)D concentration > 100 nmol/L and < 250 nmol/L, systolic BP: coefficient = −0.07, *p* <0.001; diastolic BP: Coefficient = −0.1, *p* <0.001)) [26] and reduction in all-cause mortality (25(OH)D concentration >75 nmol/L compared with 22.5 nmol/L, HR = 1.9, (95 % CI =1.6–2.2; *p* <0.001)) [27]. 

Besides the ongoing discussions on the recommended optional 25(OH)D level, other points need to be considered including some differences in measurement methods for 25(OH)D. The first developed assay was the DBP competitive protein binding assay [16]. Since 1985, a radioimmunoassay (RIA) has been available [16]. The major limitation of these assays is the fact that they also recognize other polar metabolites of vitamin D due to a cross reactivity with 24,25(OH)_2_D, therefore, leading to an overestimation of 25(OH)D levels [16]. Additionally, these measurement methods have significant inter-assay and inter-laboratory differences [15]. On the other hand, it has been reported that some immunoassays may underestimate total 25(OH)D when 25(OH)D_2_ constitutes an appreciable part of the total [28]. This may have an impact to cutoffs of vitamin D sufficiency and may have led to misclassification of patients in the past [29]. Today, direct quantitative measurement of 25(OH)D_2_ and 25(OH)D_3_ with the liquid chromatography tandem mass spectroscopy (LC-MS) represents the gold standard for measuring 25(OH)D serum concentration [16]. It is the aim of this paper to: (i) give an update of vitamin D status and vitamin D supply in a population with a western diet, namely, the German population, and to (ii) develop and discuss strategies to optimize them. These strategies should consider both the advantages as well as the disadvantages/risks of different approaches (increasing vitamin D status by dietary intake, by supplements, or by UVB-induced cutaneous synthesis).

## 2. Pandemic Vitamin D Deficiency: A Short Overview of the Vitamin D Status in Germany, A Country with A Western Diet

Vitamin D deficiency is very common worldwide; some authors even speak of a pandemic [5]. Germany, where data from the Robert Koch Institute (RKI) showed that 63% of people aged 1–17 years and 57.3% of people aged 18–79 years have 25(OH)D serum levels below 50 nmol/L (20 ng/mL) (see Table 1) [30], can serve as an example of a well-investigated population with a western diet. In line with these findings, an investigation in the Federal Republic of Germany from 2008 by Hintzpeter et al. analyzed subsample data from the German National Health Interview and Examination Survey 1998 (GNHIES) (2267 women and 1763 men) [31]. They showed that even in the sunny months (May to October), 45.2% of all men and 54.8% of all women were vitamin D deficient (25(OH)D serum level of 50 nmol/L (20 ng/mL) or below) [31]. Between November and April, 68.2% of all men and 60.8% of all women were vitamin D deficient [31]. 25(OH)D levels were measured by using a chemiluminescence immunoassay (CLIA) [31]. 

A more recent investigation based on data from the ‘German Health Interview and Examination Survey for Adults’ (DEGS1) (3635 women and 3360 men) showed that in the winter months, 25% of German men (between November and April) and 25% of German women (between November and May) had 25(OH)D serum levels <30 nmol/L [32]. Serum 25(OH)D was measured by a chemiluminescence immunoassay [32]. 

Vitamin D status in elderly people is generally lower than that in younger people [33]. Diekmann et al. investigated the vitamin D status of residents in a German nursing home in Nürnberg (84 women and 31 men) [33]. They found vitamin D deficiency (<50 nmol/L) in 93.9% of the residents [33]. This can be explained by several reasons, including the fact that residents of nursing homes have limited capacities to engage in outdoor activities and that the skin in older individuals has a reduced ability to synthesize vitamin D_3_ after exposure to UVR [33]; In that study, 25(OH)D was analyzed by an enzyme-linked immunosorbent assay (ELISA) [33]. 

Saeglitz analyzed hospitalized geriatric patients (142 women and 83 men; mean age 83 ± 5 years) in Bonn. At hospitalization, the mean serum 25(OH)D concentration was 27.4 nmol/L [34]. Of these patients, 43.9% had a serum 25(OH)D concentration below 25 nmol/L [34,35]. 25(OH)D was measured by an ^125^I radioimmunoassay [34].

Data from the DEVID study (De Vitamin in Deutschland; 1258 individuals) indicate that 25% of the participants had a 25(OH)D serum concentration below 25 nmol/L and 50% had a serum concentration between 25 and 50 nmol/L. The results for highly aged individuals were even worse: 38% of participants aged 75 years or older showed 25(OH)D serum concentrations below 25 nmol/L, whereas only 22% of participants aged 25–45 had 25(OH)D serum concentrations below 25 nmol/L [35,36]. Serum 25(OH)D was measured by a chemiluminescence immunoassay [36]. 

Another investigation showed that Turkish descendants living in Berlin have an increased risk of developing vitamin D deficiency [37]. In that study, 94.8% of women and 95.2% of men of Turkish descent had 25(OH)D serum concentrations below 50 nmol/L, compared to 86.9% of women and 78.4% of men in the group of individuals without Turkish descent [37]. In total, 597 persons of Turkish descent and 129 Berliners without Turkish descent were analyzed [37]. Serum 25(OH)D was measured by a chemiluminescence immunoassay [37]. It has been discussed that people of Turkish descent living in Germany have distinct risk factors for developing vitamin D deficiency, including relatively dark skin and wearing long clothing [37].

Concerning vitamin D status in Germany among children and adolescents, Hintzpeter et al. analyzed in the KiGGS (German National Health Interview and Examination Survey for Children and Adolescents) study (17,641 participants aged 0–17 years) [38]. 25(OH)D values were measured by using a chemiluminescence immunoassay [38]. The authors demonstrated that 31.2% of boys and 36.6% of girls aged 1–2 years with a nonimmigrant background were vitamin D deficient (25(OH)D <50 nmol/L) [38]. In the age group of 3–17 years, 64.8% of boys and 63.4% of girls with a nonimmigrant background showed vitamin D deficiency [38]. Furthermore, they analyzed the risk for vitamin D deficiency in children with different immigrant background: Boys with an Turkish or Arabic–Islamic immigrant background had an increased risk of having moderate or severe 25(OH)D concentrations (<25 nmol/L) compared with nonimmigrants ([OR] 2.3; [95% CI] 1.4–3.8 and OR 7.6; [95% CI] 3.0–19.1), whereas girls with a Turkish (OR 5.2; [95% CI] 2.9–9.6), Arab–Islamic (OR 5.9; [95% CI] 2.5–14.0), Asian (OR 6.7; [95% CI] 2.2–19.8) or African (OR 7.8; [95% CI] 1.5–40.8) background had an increased risk of having moderate or severe 25(OH)D concentrations [38].

Taken together, these data underline the importance of developing strategies to improve vitamin D status.

## 3. The Human Vitamin D Endocrine System (VDES): Molecular Biology of an Ancient Friend, Revisited

Because, in contrast to classical vitamins, vitamin D and its active metabolites are synthesized in the human body (depending on exposure of the skin to UVR), they represent a prohormone/hormone and not a vitamin [3,39].

In human skin cells, the initial substrate of cutaneous vitamin D synthesis is 7-dehydrocholesterol (7-DHC or provitamin D_3_) [6,39]. After exposure to solar or artificial UVB rays, the B-ring of the sterane structure of the 7-DHC molecule is split between atoms C9 and C10 [40]. This reaction is called photochemical conversion or photolysis [41]. The maximum wavelength leading to this photolysis is in the range of UVB radiation, i.e., approximately in the wavelength range between 290 and 310 nm [40]. By absorbing UVR photons, the 7-DHC molecule is converted into previtamin D_3_ [40]. After photolysis, previtamin D_3_ is released into the extracellular space due to altered hydrophobic and hydrophilic interactions with the fatty acids within the cell membrane [40]. Since previtamin D_3_ is energetically unstable, it spontaneously reacts to form vitamin D_3_ via a thermal isomerization reaction [40]. (For a schematic representation of the vitamin D synthesis, see Holick et al. [42].) The photolysis of 7-DHC to the product vitamin D_3_ depends on many factors (see section below).

Vitamin D_3_ is then released into the blood circulation [42]. Because of its lipophilic molecular structure, vitamin D_3_ must be bound to transport proteins [42]. The main transport protein is the vitamin D-binding protein (DBP or group-specific component (GC)-globulin), a 52–59 kDa plasma protein produced in the liver [43]. Bound in the blood to DBP, vitamin D_3_ is then transported to the liver, where it is hydroxylated by the microsomal enzyme CYP2R1 at the C-25 atom [44]. The resulting 25-hydroxyvitamin D_3_ (25(OH)D_3_ or calcidiol) is the major circulating form of vitamin D with a half-life of approximately 2–3 weeks [16]. It is well accepted that serum 25(OH)D is the best parameter for analyzing a person’s vitamin D status [16]. While the majority of 25(OH)D_3_ in the blood is bound to DBP [42], some authors suggest that 25(OH)D_3_ can be rarely (approx. 0.1%) found in an unbound, free form in serum (free hormone hypothesis) [45]. It has been discussed that this unbound 25(OH)D may drive many of the non-classical actions of vitamin D [45]. 

The complex of circulating DBP and 25(OH)D_3_ is then filtered by the kidney’s glomeruli because of its low molecular weight [46,47]. Upon filtration, the megalin/cubulin receptor complex located in the luminal membrane of renal proximal tubule epithelial cells binds the DBP/25(OH)D_3_ complex and is internalized via endocytosis into the proximal tubule cells, in a process similar to albumin and low molecular weight proteins [46,47]. Within renal cells, the complex is delivered to the lysosomal compartment, and 25(OH)D_3_ can be released into the cytosol by degrading DBP [46]. Thereafter, the 25(OH)D_3_ molecule is hydroxylated by the enzyme CYP27B1 (25-hydroxyvitamin D-1α-OHase) at the C-1 position of the molecule [42]. The resulting 1,25-dihydroxyvitamin D_3_ (calcitriol, 1,25(OH)_2_D_3_ or 1,25(OH)_2_D) is the major active form of vitamin D in the circulation [42]. In contrast to 25(OH)D, the half-life of circulating 1,25(OH)_2_D is only 4–6 h, and the serum levels of 1,25(OH)_2_D are a thousand-fold lower compared to 25(OH)D [16].

After binding to DBP, 1,25(OH)_2_D_3_ produced in the kidneys is transported via the circulation to distinct target organs, predominantly to tissues involved in bone and calcium metabolism [48]. However, many tissues not involved in bone and calcium metabolism are also target organs for biologically active vitamin D metabolites [45]. Many of these extrarenal cells, for example, immune cells, including monocytes, macrophages and dendritic cells, also express CYP27B1, possess α-hydroxylase activity and produce 1,25(OH)_2_D_3_ locally [45]. It has been discussed that this extra-renally produced 1,25(OH)_2_D_3_ is not transferred to the circulation as an endocrine hormone but exerts regulatory effects locally via autocrine or paracrine effects in various tissue-dependent cellular functions [45]. In target cells, 1,25(OH)_2_D_3_ exerts many of its pleiotropic genomic effects via binding to the nuclear vitamin D receptor (VDR) [48]. The VDR is a nuclear receptor that, after forming a heterodimer with the retinoid-X receptor (RXR), regulates the transcriptional activity of target genes via binding to specific DNA recognition sequences called response elements [14]. It has been estimated that 1,25(OH)_2_D_3_ regulates more than 200 genes by these mechanisms [14].

In addition to the traditional pathway of vitamin D activation via 7-DHC, 25(OH)D and 1,25(OH)_2_D, other activation pathways with alternative metabolites, including 20(OH)D and 20,22(OH)_2_D, have been reported [49].

A key role in this pathway is cytochrome P450 side-chain cleavage (P450scc), encoded by the CYP11A1 gene [50]. P450scc catalyzes a three-step reaction: first, the 22-hydroxylation of cholesterol; second, the 20-hydroxylation of 22(R)-hydroxycholesterol; and finally, the oxidative scission (bond cleavage) between atoms C20 and 22 of the 20(R),22(R)-dihydroxycholesterol molecule [51]. This reaction is called a side-chain cleavage event resulting in the end product pregnenolone, a precursor of steroid hormones [51]. During the reactions, the metabolites do not leave the active site of CYP11A1 [52].

Scientific findings indicate that CYP11A1 is important for VDES by catalyzing 7-DHC to 7-dehydropregnenolon in a three-step reaction (with the intermediate metabolites 22(OH)7-DHC and 20,22(OH)_2_7-DHC) analogous to the reaction of cholesterol to pregnenolone [52,53].

Additionally, it has been reported that CYP11A1 hydroxylates previtamin D_3_ to the secosteroid 20(OH)D_3_ and finally to 20,22(OH)_2_D_3_ [52]. Notably, it has been shown that the nuclear aryl hydrocarbon receptor (AhR) is the major corresponding receptor target for 20,23(OH)2D_3_ [9].

However, most of the non-skeletal effects of vitamin D remain highly controversial [54]. 

## 4. How Can a Healthy Vitamin D Status Be Achieved and Maintained? Relevance of Supplements and Dietary Intake

In addition to UVB-induced vitamin D production in the skin, in humans, there are two other possible sources of vitamin D: diet and supplements. In the absence of endogenous synthesis of vitamin D, the present guidelines of the German society for nutrition (Deutsche Gesellschaft für Ernährung (DGE) e.V.) and the D-A-CH society (i.e., Germany, Austria, Switzerland) recommend an intake of 400 IU (10 µg) vitamin D per day for infants and 800 IU (20 µg) per day for children, adolescents, adults, pregnant women and breast-feeding women [55]. Previously, the D-A-CH society recommended a lower vitamin D intake, namely, 200 IU (5 µg) per day for younger people and 400 IU (10 µg) per day for persons older than 65, respectively [56]. 

In addition to vitamin D intake with breastmilk or infant food, German pediatricians recommend an oral supplementation with 400–500 IU vitamin D_3_ per day for all infants up to the second summer (duration from 1–1.5 year, depending on birth date) [57]. Premiees with a birth weight below 1500 g should be given 800–1000 IU vitamin D daily during the first months of life [57]. 

The current Endocrine Society clinical practice guideline suggests for children aged 0–1 year at least 400 IU per day of vitamin D, for children from 1 year, adults aged 19–70, pregnant and lactating women at least 600 IU per day of vitamin D and for adults from aged 70 years 800 IU per day of vitamin D [18]. However, to raise the blood level of 25(OH)D above 75 nmol/L (30 ng/mL), higher intakes of vitamin D have been recommended (1000 IU per day for children and 1500–2000 IU per day for adults) [18]. For obese children and adults and adults on anticonvulsant medications, glucocorticoids, antifungals and medication for AIDS, the Endocrine Society suggests at least two to three times more vitamin D for their age group [18]. 

Concerning vitamin D deficiency, the Endocrine Society suggests that all adults who are vitamin D deficient should be treated with 50,000 IU of vitamin D once a week for 8 weeks or its equivalent of 6000 IU of vitamin D daily to achieve a blood level of 25(OH)D above 75 nmol/L (30 ng/mL), followed by maintenance therapy of 1500–2000 IU per day [18]. For children aged 0–18 years who are vitamin D deficient, the Endocrine Society recommends treatment with 2000 IU per day of vitamin D or with 50,000 IU of vitamin D once weekly for 6 weeks to achieve a blood level of 25(OH)D above 75 nmol/L (30 ng/mL), followed by maintenance therapy of 400–1000 IU per day (for infants aged 0–1 year) or 600–1000 IU per day (for children aged 1–18 years), respectively [18]. For obese patients, patients with malabsorption syndromes and patients on medications affecting vitamin D metabolism, the Endocrine Society suggests two to three higher doses of vitamin D to treat vitamin D deficiency, followed by a maintenance therapy of 3000–6000 IU per day [18].

On the other hand, the Institute of Medicine (IOM) has defined a tolerable upper intake level for vitamin D at 1000 IU per day in infants ages 0–6 months, 1500 IU per day in infants ages 6–12 months, 2500 IU per day in children ages 1–3 years, 3000 IU per day in children ages 4–8 years and 4000 IU per day in adolescents and adults [20,58]. However, the Endocrine Society clinical practice guidelines noted that higher dosages may be needed to correct vitamin D deficiency [18]. 

### 4.1. Ingestion, Absorption and Bioavailability of Vitamin D

Vitamin D from diet or supplements may be present as vitamin D_3_ (cholecalciferol) or vitamin D_2_ (ergocalciferol) [42,59,60]. Non-hydroxylated vitamin D is a lipophilic molecule that is closely related to cholesterol [60,61]. In the intestinal lumen, other lipids (such as triglycerides or phospholipids) are important for solubilizing fat-soluble nutrients in micelles and for their absorption [60]. Digestive enzymes catalyze the release of monoglycerides and phospholipids from lipids, resulting in the formation of further micelles [60]. However, there are no consistent data to support the hypothesis that a diet containing high amounts of fat may improve the bioavailability of vitamin D [60], although some data suggest that the type of dietary fat is important: some investigators have found that a high consumption of monounsaturated fatty acid-rich oils may improve the bioavailability of supplemental vitamin D_3_ [62]. Additionally, there are published data indicating that dietary fiber, vitamin E, vitamin A, phytosterols and plant sterols may reduce the bioavailability of vitamin D [60]. However, the precise underlying mechanisms as well as the role of other possible factors influencing vitamin D bioavailability, such as gastric pH or the role of intestinal enzymes, are not completely understood [60].

Depending on the concentration of vitamin D, two different mechanisms of absorption in the gastro-intestinal system have been reported [60] if present in low concentrations (i.e., vitamin D in the diet is often weakly concentrated), transport through the enterocyte cell membrane is mediated by distinct specific proteins [60]. It has been suggested that proteins involved in protein-mediated transport may include SRBI (scavenge receptor class B type 1), CD 36 (cluster determinant 36) and NPC1L1 (Neimann–Pick C1-Like 1) [60,61]. Uptake of vitamin D in high concentrations, usually resulting from intake of supplements, is mediated via passive diffusion through the enterocyte cell membrane [60]. These mechanisms may explain the reported fact that vitamin D from diet has only an estimated 60% bioavailability compared to vitamin D supplementation [60]. 

The human diet also contains hydroxylated metabolites of vitamin D (25(OH)D_2_ and 25(OH)D_3_) (Table 2) [60]. The absorption efficiency and thereby the bioavailability of hydroxylated metabolites of vitamin D has been reported to be up to ten times higher than the bioavailability of non-hydroxylated forms, but little is known about the specific underlying mechanisms [60]. 

After ingestion with food or supplements and absorption in the gut, both vitamin D_2_ and vitamin D_3_ are transported via chylomicrons and DBP via the lymphatic system to the liver [63,64], where they are converted into their analogous metabolites, namely, 25(OH)D_2_ and 25(OH)D_3_, similar to cutaneously produced vitamin D [63]. Finally, 25(OH)D produced in the liver enters the blood circulation, where it binds to DBP and lipoproteins [42].

### 4.2. The Role of Vitamin D_2_

Only plants and fungi possess ergosterol in their cell membranes; therefore, they are the only ones able to produce vitamin D_2_ forms [40]. Although vitamin D_2_ and D_3_ molecules are quite similar, there are some differences in their structural formulas. Vitamin D_3_ has a molar mass of 384, while vitamin D_2_ has a molar mass of 396 [65]. Investigations have shown that vitamin D_2_ metabolites bind with a lower affinity to DBP compared with vitamin D_3_ metabolites, resulting in a relative inefficiency of vitamin D_2_ to raise total serum 25(OH)D levels, as well as a shorter plasma half-life and a lower activity of D_2_ metabolites [45]. Because of the low binding affinity, 25(OH)D_2_ is also transported in chylomicrons and lipoprotein fraction 3 (LPF3). In contrast to DBP, chylomicrons and LPF3 fractions are not able to protect bound molecules against degradation or excretion [64]. It has been reported that in addition to the traditional pathway of ergosterol (similar to vitamin D produced in the skin), ergosterol can be hydroxylated by CYP11A1, first at the C24 position and second at C17, without side chain cleavage, resulting in 17α,24-dihydroxyergosterol (17α,24(OH)_2_D_2_) [66]. Many other alternative substrates, such as 20-hydroxy-22,23-epoxy-22,23dihydroergosterol and 22-keto-23-hydroxy-22,23-dihydroergosterol, have been described [67]. 

It is well accepted that vitamin D_3_ is more effective at raising total 25(OH)D concentrations than vitamin D_2_ [68,69,70,71,72]. Some investigations have shown that vitamin D_2_ intake increases 25(OH)D_2_ but produces a decline in 25(OH)D_3_ [68,69,70]. Recently, Martineau et al. reported that administration of vitamin D_2_ reduces 25-hydroxylation of vitamin D_3_ and 1-alpha hydroxylation of 25(OH)D_3_, while increasing 24R-hydroxylation of 25(OH)D_3_ [72]. Vice versa, it has been reported that vitamin D_3_ intake is associated with a decrease in 25(OH)D_2_ level, suggesting a common regulatory mechanism [73]. However, the exact mechanisms are unknown [73]. 

Armas et al. treated 20 healthy male volunteers with a single dose of 50,000 IU of vitamin D_2_ or D_3_ [69]. They showed a similar initial increase in serum 25(OH)D over the first 3 days in both groups. But only in the D_3_-treated subjects 25(OH)D level continued to rise, whereas serum 25(OH)D level fell rapidly in the D_2_-treated subjects and was not different from baseline after two weeks [69]. In an umbrella review of systematic reviews and meta-analyses of observational studies and randomized trials done by Theodoratou et al., it has been reported that vitamin D_2_ supplementation is associated with increased (but non-significant) risk of mortality (reported summary effect: 1.04 (CI 0.97 to 1.11), *p*-value: 0.26) [74].

### 4.3. Vitamin D from Supplements

In Germany, many oral vitamin D supplements containing ergocalciferol or cholecalciferol (and with or without additional components such as calcium carbonate) are easily available and are accessible over the counter without a physician’s prescription. Higher dosed oral supplements (from 5600 IU) as well as hydroxylated vitamin D derivates (alfacalcidiol, paricalcitol or calcitriol) are available only on prescription. 

The German federal healthy survey 1998 (BGS) done by the Robert Koch Institute analyzed vitamin supplementation of 7124 participants aged 18–79 (response rate: 61%) [75]. The investigators found that 57 participants used oral vitamin D supplementation every day. The mean daily vitamin D intake was 292 IU (7.4 µg) [75]. According to the results of the German national consumption study (Nationale Verzehrstudie) from 2008 (8278 women and 7093 men), 477 women and 270 men used vitamin D supplements [56]. The mean intake of vitamin D was 200 IU (5 µg) per day for women and men [56]. Vitamin D supplementation in women increased with increasing age, whereas the highest vitamin D supplementation in men was in the age from 35 to 50 [56]. 

Several studies analyzed vitamin D supplementation in terms of dosage, administration and efficacy to increase 25(OH)D level. Direct comparisons between oral and intramuscular supplementation of vitamin D_3_ showed that both effectively increase serum 25(OH)D concentrations [76,77].

Garland et al. analyzed the increase in 25(OH)D serum concentration after vitamin D supplementation [78]. They estimated that starting at baseline 25(OH)D serum concentrations of 25 nmol/L (10 ng/mL), 75 nmol/L (30 ng/mL), 125 nmol/L (50 ng/mL) and 225 nmol/L (90 ng/mL), oral uptake of 1000 IU per day may result in mean increases in 25(OH)D serum concentrations of 27.5 nmol/L (11 ng/mL), 20 nmol/L (8 ng/mL), 12.5 nmol/L (5 ng/mL) and 4 nmol/L (1.6 ng/mL), respectively [78]. Multivariable regression analysis by Zittermann et al. showed that vitamin D dose per kg body weight per day could explain 34.5% of variation in circulating 25(OH)D [79]. Other factors influencing the 25(OH)D level after supplementation were type of supplement (D_2_ or D_3_), age, concomitant intake of calcium supplements and baseline 25(OH)D [79]. 

A recent study randomized 60 subjects with vitamin D deficiency in Belgium who were either treated with 2000 IU vitamin D_3_ orally per day or 50,000 IU orally per month (cumulative dose: 150,000 IU vitamin D_3_ in each treatment group) [80]. The authors showed that the monthly supplementation was an effective tool for a rapid normalization of 25(OH)D_3_ in deficient subjects, whereas the daily administration was similarly effective but took two weeks longer to reach the desirable level of 50 nmol/L (20 ng/mL) [80]. Kearns et al. investigated in a meta-analysis review the efficacy of a single large bolus dose for the treatment of vitamin D deficiency [81]. They concluded that a single dose of vitamin D_3_ ≥300,000 IU may be effective to improve vitamin D status for up to 3 months [81]. A more recent investigation showed that 50,000 IU monthly and 150,000 IU 3-monthly of vitamin D_3_ safely and effectively correct vitamin D deficiency in adolescents [82]. Takacs et al. compared the efficacy of daily, weekly and monthly administration of vitamin D_3_ [83]. They concluded that the daily, weekly and monthly administrations of daily equivalent of 1000 IU of vitamin D_3_ provide equal efficacy and safety profiles [83]. 

However, it has to be noted that high-doses supplementation, as mentioned Kearns et al. [81], among others, may be harmful and increases the risk of intoxication. Rossini et al. reported an extremely high 25(OH)D peak increment to 167.75 nmol/L ± 42.76 nmol/L (67.1 ± 17.1 ng/mL, *p* <0.001) three days after a single oral bolus of 600,000 IU vitamin D_3_ [84]. Symptoms of vitamin D toxicity include hypercalcemia, hypercalciuria, kidney stones, hyperphosphatemia, polyuria, polydipsia, ectopic calcification of soft tissues, nausea/vomiting, anorexia, constipation, headache and hypertension [81,85,86,87,88]. Hypercalciuria has been reported to be a more sensitive criterion for vitamin D excess than hypercalcemia [89]. Kahawati et al. reported in a systematic review (3 RCTs [n = 39,213]) that supplementation using vitamin D with calcium (1000 IU vitamin D_3_/1400 mg calcium daily, 2000 IU vitamin D_3_/1500 mg calcium daily and 400 IU vitamin D_3_/1000 calcium daily, respectively) was associated with an increased incidence of kidney stones (pooled absolute risk differences (ARD), 0.33% [95% CI, 0.06% to 0.60%]) [90]. Thus, those taking high vitamin D doses should reduce or eliminate calcium supplementation. However, in an investigation by Ferraro et al., vitamin D intake in typical amounts was not statistically associated with risk of kidney stone formation [91]. They also did not observe a higher risk, even among those participants with higher intakes of calcium [91]. In a recent systematic review analysis (15 studies, 3150 participants, ≥2800 IU per day vitamin D_2_ or D_3_ supplementation), Malihi et al. demonstrated that one year or longer supplementation with a daily, weekly or monthly dose of vitamin D_2_/D_3_ did not significantly increase the risk of total adverse events or kidney stones, although there was a trend towards increased hypercalcemia, and possibly for hypercalciuria [92]. 

Besides supplementation with cholecalciferol and ergocalciferol, hydroxylated vitamin D derivates (in Germany: alfacalcidiol, calcitriol, paricalcitol) are available that can be administered orally or intravenously. A review analysis by Mazess et al. (21 clinical trials that compared intravenous and oral vitamin D analogs (calcitriol, alfacalcidol, doxercalciferol) for the treatment of secondary hyperparathyroidism in hemodialysis patients), the authors found no difference in efficacy between intravenous and oral vitamin D hormone supplementation [93]. 

### 4.4. Vitamin D from Diet

Only a few foods naturally contain vitamin D or 25(OH)D_3_ in substantial amounts (Table 2 and Table 3) [14,94,95]. 

For example, 100 g of fresh wild salmon contains approximately 600–1000 IU of vitamin D [14]. One hundred grams of fresh shiitake mushrooms contains approximately 100 IU of vitamin D, and an egg yolk contains approximately 20 IU of vitamin D [14]. The content of vitamin D may differ from the origin of the product [94].

Meat may also be a source of vitamin D [95]. In an investigation by Crowe et al., plasma 25(OH)D levels were analyzed in meat eaters, fish-eaters and vegetarians (1388 meat-eaters, 210 fish-eaters, 420 vegetarians and 96 vegans, aged 20–76 years from the European Prospective Investigation into Cancer and Nutrition (EPIC)–Oxford cohort) [96]. The authors found that meat-eaters had the highest mean intake of vitamin D (124 IU, 3.1 (95% CI 3.0, 3.2) µg per day) and mean plasma 25(OH)D concentrations (77.0 (95% CI 75.4, 78.8) nmol/L) and vegans the lowest (28 IU, 0.7 (95% CI 0.6, 0.8) µg per day and 55.8 (95% CI 51.0, 61.0) nmol/L, respectively [96].

In Germany, the main source of foods with a considerable amount of vitamin D is fish or crustacean representing over one-third of vitamin D intake, followed by fish-based dishes representing 15% of all consumed vitamin D products [56]. Approximately 10% of vitamin D intake comes from fats, eggs and milk/cheese [56]. On average, a German man consumes 29 g of fish, fish products, crustaceans and related dishes and a German woman 23 g per day [56]. In a study with 19 European countries (including Germany) ocean fish was the most important single dietary factor affecting serum 25(OH)D concentration for postmenopausal women, but animal fat and meat also contributed [97,98].

According to the results of the German national consumption study (Nationale Verzehrstudie), the median vitamin D intake for men is 116 IU (2.9 µg) per day and for women is 88 IU (2.2 µg) per day. The median intake of vitamin D increases in men and women up to the age of 51–64 years and remains constant at ages above that age group [56]. The mean intake of vitamin D at the age of 65 years or older is 132 IU (3.3 µg) for men and 88 IU (2.2 µg) for women [56]. Based on the previously recommended vitamin D intake of the D-A-CH society (200 IU per day and 400 IU per day for persons older than 65, respectively), the National Consumption Study calculated that a total of 82% of men and 91% of women did not achieve the recommended daily intake of vitamin D [56]. 

Regarding children and young people, the vitamin D intake is even lower: according to the EsKiMo study (nourishing module of the KiGGS study; 1258 girls and 1248 boys), the median vitamin D intake of 6-year-old boys is 56 IU (1.4 μg), and girls of the same age consume 52 IU (1.3 μg) per day as the median. Boys aged 15–17 years have a median daily vitamin D intake of 100 IU (2.5 μg) and girls in this age group of 68 IU (1.7 μg) [99]. 

It has to be noted, that in addition to the relatively low vitamin D content of most foods, some other factors may limit oral vitamin D uptake, including malabsorption in the gastrointestinal tract (e.g., resulting from cystic fibrosis, celiac disease, Whipple’s disease, Crohn’s disease, bypass surgery and/or medications such as lipid-lowering agents) [14,61].

### 4.5. Vitamin D Food Fortification

Because most foods contain little vitamin D, there is mounting support for vitamin D fortification of common foods [100,101,102,103]. For the USA and Canada, it has been estimated that approx. 60% of the intake of vitamin D from foods comes from fortified foods [104]. In Finland, Jääskeläinen et al. showed that after starting vitamin D fortification of fluid milk products and fat spreads in 2003, the vitamin D status of Finnish adults has improved considerably [105]. In Denmark, Grønborg et al. found that vitamin D-fortified foods (yoghurt, cheese, eggs and crisp bread) improved vitamin D status of women of Danish and Pakistani origin during winter [106]. The same group analyzed different scenarios increasing vitamin D intake in Danish women (diet without fish, diet including fish, fortified foods and supplements) [107]. They concluded that low-dose fortification of different foods with vitamin D may be an effective and safe population-based approach [107]. Outcomes of the ODIN project (food-based solutions for optimal vitamin D nutrition and health through the life cycle) showed that vitamin D-food fortification safely increases population intakes and prevents low vitamin D status [108]. Keller et al. analyzed the risk of development of gestational diabetes of Danish women who were exposed during their fetal life to extra vitamin D from food fortification [109]. They demonstrated that prenatal exposure to extra vitamin D from mandatory fortification may lower the risk of developing gestational diabetes among spring-born women [109]. Besides of conventional vitamin D fortification as a nutrient supplement, there are some novel approaches to vitamin D enrichment of food, including “bio-additions” (ex. the exposure of edible mushrooms to UVR) and bio-fortification (enhancing nutritional quality through agronomic or modern biotechnology techniques) [68,104].

However, in Germany, vitamin D food fortification is still limited to margarine, based on a law of 1942 [100].

Taken together, since little food contains sufficient vitamin D and food fortification does not play a role in Germany due to legal requirements, vitamin D supplementation is a promising strategy for increasing vitamin D levels according to the recommendations of the guidelines. However, the upper intake levels should not be exceeded for a long time and physicians and patients should be aware of symptoms of intoxication.

## 5. Vitamin D Status in Humans: Relevance of UVB-induced Cutaneous Vitamin D Production

Since vertebrates like humans do not have ergosterol in their cell membrane, they produce vitamin D_3_ exclusively in their skin [40]. Due to the ubiquitous availability of solar UVR in most regions worldwide, especially during spring and summer, and because most foods contain little vitamin D, cutaneous vitamin D production is developed in humans as the most important source for fulfilling the human body’s requirements for vitamin D. On the other hand, populations such as Greenland´s indigenous people with dark skin pigmentation have a diet that is predominantly based on marine mammals containing large amounts of vitamin D [1,2,3,4,110].

The UVB-induced cutaneous vitamin D production depends on many individual factors. For example, Holick et al. showed that vitamin D_3_ production in Boston, located at the 42° N latitude in the US state of Massachusetts, is only possible between March and November [5]. In July, vitamin D photosynthesis begins between 9 and 10 a.m. and ends around 6 p.m. in the evening [5]. In contrast to Boston, vitamin D_3_ production in Bergen (Norway), which is 18° north of Boston, is only possible between April and October and within a time period between 11 a.m. and 6 p.m. in July [5]. Similar results were shown by Webb et al. [111]. They found that in Boston, vitamin D production is possible from November through February [111]. In Edmonton (10° north of Boston), in the Canadian province of Alberta, the photosynthesis of previtamin D_3_ stops from October to April [111]. Germany is located between the latitude of Boston and Edmonton, specifically between 47° N and 55° N latitude [32]. The dependence on latitude, season and time of day is well explained by the fact that the path of UVB through the atmosphere depends on the solar zenith angle. This path is longer during winter months, at higher latitudes and during morning and evening hours, resulting in an increased absorption of UVB photons by ozone molecules in the stratosphere that leads to reduced numbers of UVB photons reaching the earth’s surface–and the skin [5,40]. Effects of climate change cause complex changes in stratospheric ozone. In some regions these changes will enhance levels of UVR, while in others they will reduce UVR [112]. 

In addition to these geographical factors, many other factors have a complex impact on cutaneous vitamin D synthesis, including season, whether conditions, time of exposure [5], clouds and aerosols in the air [113], skin pigmentation based on the genetic background [14,114,115], body fat mass [116], age [40], UVR-exposed body surface area [117], medication, and liver or kidney diseases [14]. In a systematic review by Xiang et al., the authors conclude that pigmented skin has less effective photoproduction of vitamin D and 25(OH)D following experimental UVR exposure [118]. However, an investigation by Hakim et al. with Caucasian women (skin types II–V) found no skin type or ethnicity-dependent differences in production of 25(OH)D after identical UVR exposure [119].

Older experimental data indicate that the use of sunscreen suppresses cutaneous vitamin D synthesis [120]. However, a recent investigation showed that the use of sunscreens with sun protection factor (SPF) 15 and a high UVA-protection factor applied at sufficient thickness still allows a highly significant improvement of serum 25(OH)D concentration [121]. These results are in accordance with a recent review showing that sunscreen use for daily and recreational photoprotection does not compromise vitamin D synthesis [122]. 

GWAS analysis by Wang et al. identified variants near genes involved in cholesterol synthesis (DHCR7), hydroxylation (CYP2R1, CYP24A1), and vitamin D transport (*GC*) that influence vitamin D status [43]. 

Interestingly, a longer UVR exposure of the skin does not necessarily provide more vitamin D; instead, the level may even decrease via two different mechanisms: photochemical regulation and photodegradation. Along with a higher risk of sunburn and skin cancer, prolonged UVR or sun exposure is therefore not associated with an additional health benefit. On the other hand, photochemical regulation and photodegradation prevent excessive vitamin D production and the development of vitamin D intoxication via cutaneous synthesis [123]. During prolonged exposure to the sun or at excessively high wavelengths of UVR, inactive byproducts of previtamin D_3_ are formed: Previtamin D_3_ is reversibly converted into photoisomers tachysterol 3 and lumisterol 3 and finally irreversibly into toxisterols [40,124]. A similar effect is due to the second mechanism, called photodegradation of vitamin D_3_ [125]. With increasing wavelength before being released into the blood circulation, vitamin D_3_ is irreversibly converted via a photolysis reaction into three possible products: 5,6-trans vitamin D_3_, suprasterol 1 and suprasterol 2 [125].

Investigations have shown that a single whole-body UVB irradiation with 1 minimal erythema dose (MED) corresponds to an oral intake between 10,000 and 25,000 IU of vitamin D_2_ [40]. The UVR exposure of ¼ MED on ¼ of skin area (hands, face and arms) is equivalent to a dietary intake of about 1000 IU vitamin D [126]. The MED is defined as the amount of UVR that will produce minimal erythema (redness caused, e.g., by dilated capillaries) of an individual’s skin within a few hours following exposure [127]. Chen et al. measured the serum concentrations of 25(OH)D in 15 healthy young adults (skin types II and III, age 20–53 years) after most parts of their bodies were exposed three times a week (0.75 MED per week) for a period of 7 weeks. The mean total dose of UVB radiation was approximately 4 MED. They found a 50% increase in the baseline serum 25(OH)D level after one week. This increase continued for five weeks before reaching a plateau of approximately 150% above baseline values [40]. However, when repeating this investigation with a 76-year-old male, his serum 25(OH)D reached a plateau after 2 weeks of exposure, but at only approximately 60% above baseline values [40]. 

More recent investigations use the standard erythema dose (SED) [128]. One SED is equivalent to an erythemal exposure of 100 J/m^2^ [128]. The expected MEDs of skin types I to IV is in the region of 2.5–4.5 SED on previously unexposed buttock [129]. Rhodes et al showed that 13 min of midday sunlight exposure on a cloudless day, three times weekly, to 35% skin surface area over a 6-week summer period was sufficient to achieve in 90% of the participants (120 volunteers, white Caucasians, sun-reactive skin types I–IV, aged 20–60 years, from Greater Manchester, UK) the vitamin D sufficiency range (25(OH)D ≥ 50 nmol/L) [130]. However, only a minor portion of the participants (26%) reached vitamin D levels in the proposed optimal range (25(OH)D ≥ 80 nmol/L) [130]. Manchester shares latitude with northern parts of Germany. Webb et al. analyzed these results under real-life conditions [131]. After a mean daily midday exposure of 9 min (weekdays) and 18 min (weekends) at the end of the summer, the mean increase in 25(OH)D was 71 nmol/L [131]. The authors concluded that relatively short, frequent exposures to sunlight are effective to increase vitamin D status [131]. Narbutt et al. analyzed changes in 25(OH)D concentration after UVR of 32 healthy Polish children (skin types I–IV) during a holiday on the Baltic Sea [132]. Poland is the eastern neighbor of Germany with similar latitude. The investigators found that after daily UVR exposure (in average 2.4 ± 1.5 SEDs) the mean concentration of 25(OH)D_3_ increased (×1.24 ± 0.19) from 64.7 ± 13.3 to 79.3 ± 18.7 nmol/L (*p* <0.001) [132]. 

It has to be noted that there are other positive UVB-induced actions of sunlight: in addition to 7-dehydrocholesterol, UVB photons can be absorbed by a range of chromophores, including DNA, membrane lipids, urocanic acid with subsequent effects on immune cells and secretion of neuropeptides such as β-endorphin and α-melanocyte stimulating hormone (MSH) [133,134]. On the other hand, it must be considered that patients with photodermatoses such as erythropoietic protoporphyria, for example, have to avoid solar or artificial UVR [135]. Consequently, vitamin D deficiency in patients with erythropoietic protoporphyria has been reported [135]. 

In summary, it can be concluded that UVB exposure of approximately 20–25% of the body surface (e.g., arms and legs) for 5–30 min (depending on time of day, season, latitude, and skin pigmentation) between the hours of 10 a.m. and 3 p.m. for white adults, 2–3 times per week, in spring, summer and autumn represents a promising strategy to obtain and maintain a sufficient vitamin D status [3,5,14]. As mentioned above, since only a few foods naturally contain vitamin D in substantial amounts (Table 2 and Table 3), moderate UVB-exposure is an important vitamin D source for fulfilling the human body’s requirement for vitamin D. By sufficiently exposing the skin of a healthy person in spring, summer and autumn to solar UVR, no additional supply of vitamin D from the food or from supplements is necessary [39]. A longer exposure to the sun does not provide more vitamin D-associated health benefits but is associated with a higher risk of sunburn and skin cancer.

## 6. Conclusions: A Critical Appraisal of Strategies to Optimize Vitamin D Status in Germany 

To obtain and maintain a sufficient vitamin D status, a healthy German adult with skin type I–III should be exposed to the sun of approximately 20–25% of the body surface (e.g., arms and legs) for 5 to 30 min between the hours of 10 a.m. and 3 p.m., 2–3x/week, in spring, summer and autumn without reaching his or her individual MED [3,5,14]. A longer exposure to the sun does not provide more vitamin D-associated health benefits. Although oral intake of vitamin D from both food and supplements leads to the same endproducts as UVB-dependent cutaneous vitamin D production, the bioavailability of the latter is not affected by potential malabsorption in the gut, for example. On the other hand, UVB-induced cutaneous vitamin D production only results in D_3_ analogs. A comparison is shown in Table 4.

In the absence of endogenous synthesis of vitamin D, the present guidelines of the German society for nutrition (Deutsche Gesellschaft für Ernährung (DGE) e.V.) and the D-A-CH society (i.e., Germany, Austria, Switzerland) recommend an intake of 400 IU (10 µg) vitamin D per day for infants and 800 IU (20 µg) per day for children, adolescents, adults, pregnant women and breast-feeding women [55]. 

Especially during the winter and autumn months, the population should regularly eat vitamin D-rich food, such as fish dishes, along with maintaining a healthy lifestyle in general. 

Concerning vitamin D deficiency, the Endocrine Society Clinical Practice guideline suggests that all adults who are vitamin D deficient should be treated with 50,000 IU of vitamin D once a week for 8 week or its equivalent of 6000 IU of vitamin D daily to achieve a blood level of 25(OH)D above 75 nmol/L, followed by maintenance therapy of 1500–2000 IU per day [18]. However, the growing rate of vitamin D supplementation could increase costs and be harmful, especially if the tolerable upper intake level is exceeded (1000 IU per day in infants ages 0–6 months, 1500 IU per day in infants ages 6–12 months, 2500 IU per day in children ages 1–3 years, 3000 IU per day in children ages 4–8 years and 4000 IU per day in adolescents and adults [20,58]). 

In the literature, there is currently no consistency regarding whether or not to measure 25(OH)D, with present recommendations being mostly based on expert opinions, resulting in a relatively low evidence level [15]. For example, Holick defined risk groups for the development of vitamin D deficiency. These groups include breastfeeding children up to 1 year without vitamin D supplementation, children from 1 through 18 years of age with inadequate sun exposure or supplementation or dark skin, adults with inadequate sun exposure or supplementation, a decreased 7-dehydrocholesterol level in skin because of aging (over 50 years), pregnant or lactating (fetal utilization, inadequate sun exposure or supplementation) women, patients with malabsorption syndromes (malabsorption of vitamin D, inadequate sun exposure or supplementation), patients with drug intake that activates steroid and xenobiotic receptors or drugs used in transplantation, patients with obesity, patients with nephrotic syndrome, patients with a higher stage of chronic kidney disease, patients with primary or tertiary hyperparathyroidism, as well as patients with granulomatous disorders and some lymphomas [14]. In these groups, preventive and maintenance measures to avoid vitamin D deficiency are recommended. These measures include a specific oral supplementation of vitamin D products for each risk group, if necessary [14]. In the groups of adults with inadequate sun exposure or supplementation, a decreased 7-dehydrocholesterol level in skin because of aging (over 50 years) and malabsorption syndromes, Holick recommends skin exposure to artificial UVB irradiation [14]. 

Taken together, it is important for most populations that individuals regularly consume vitamin D-rich food, especially during the winter and autumn months. Individuals with the risk factors mentioned above as well as hypovitaminosis D in the past, osteoporosis, osteomalacia, dark pigmentation (skin type from III), no access to sunlight (e.g., patients confined to bed), photodermatosis with the necessity of avoiding UVR or unhealthy lifestyle in general should be monitored by measuring and supplementing vitamin D_3_ if necessary. The other part of the population as well as patients with malabsorption syndromes should be regularly moderately exposed to UVR (approximately 20–25% of the body surface (e.g., arms and legs) for 5–30 min (depending on time of day, season, latitude, and skin pigmentation) between the hours of 10 a.m. and 3 p.m., 2–3 times per week, in spring, summer and autumn) to ensure the health benefits of UVB exposure with minimal increased risk for skin cancer. 

## Figures and Tables

**Table 1:** Prevalence of 25(OH)D serum concentration in Germany, classified by age and sex (cited from reference [30]).

25(OH)D [nmol/L]	Age 1–17 Years			Age 18–79 Years		
	All (n = 10,015)	Male (n = 5107)	Female (n = 4908)	All (n = 3917)	Male (n = 1706)	Female (n = 2211)
**<12.5**	3.8%	3.6%	4.0%	2.0%	2.2%	1.9%
**12.5 to<25**	15.5%	15.6%	15.4%	14.3%	13.4%	15.1%
**25 to<50**	43.7%	42.9%	44.5%	41.0%	41.2%	40.8%
**50 to<75**	22.8%	23.3%	22.3%	20.8%	22.6%	19.1%
**>75**	14.2%	14.6%	13.8%	21.9%	20.6%	23.1%

**Table 2:** Vitamin D_3_ and 25(OH)D_3_ content of chosen foods, modified by [94,95].

Food (100 g)	Vitamin D_3_	25(OH)D_3_
**Raw eggs**	12–100 IU (0.3–2.5 µg)	0.1–1.5 µg
**Cooked eggs**	12–92 IU (0.3–2.30 µg)	0.18–1.2 µg
**Raw white fish**	4–188 IU (0.1–4.7 µg)	0.30–0.70 µg
**Cooked white fish**	4–232 IU (0.1–5.8 µg)	0.35–0.60 µg
**Beef**	4.4–5.6 IU (0.11–0.14 µg)	0.15–0.16 µg
**Lamb**	6.8–10.8 IU (0.17–0.27 µg)	0.16–0.18 µg
**Chicken**	11.6–17.6 (0.29–0.44 µg)	0.36–0.51 µg
**Pork**	7.2 IU (0.18 µg)	0.17 µg

**Table 3:** Vitamin D content of chosen foods, modified by [14].

Food	Approximate Vitamin D content
**Fresh, wild Salmon (99.05 g)**	600–1000 IU
**Fresh, farmed salmon (99.05 g)**	100–250 IU
**Canned salmon (99.05 g)**	300–600 IU
**Canned sardines (99.05 g)**	300 IU
**Canned mackerel (99.05 g)**	250 IU
**Canned Tuna (101.88 g)**	230 IU
**Cod liver (1 teaspoon)**	400–1000 IU
**Fresh shiitake mushrooms (99.05 g)**	100 IU
**Sun-dried shiitake mushrooms (99.05 g)**	1600 IU
**Egg yolk**	20 IU

**Table 4:** Comparison between cutaneous Vitamin D production and oral intake (includes food and supplementations).

Parameter	Cutaneous Vitamin D Production	Oral Intake (Food and Supplements)
**Vitamin D compounds**	Exclusively D_3_ analogues	D_3_ and D_2_ analogues
**Skin damage**	Risk of sunburn, skin cancer and actinic damage	-
**Risk of intoxication**	In healthy individuals, no risk for UVB-induced vitamin D intoxication	Risk of intoxication (only by supplements with very high doses of vitamin D)
**Accessibility**	Ubiquitously available in summer months	Low amount of vitamin D contents in food
**Limitation factors**	Many individual limitation factors such as age, pigmentation, season, geographic and meteorological factors	Reduced absorption in patients with malabsorption syndromes
**Other effects**	Other positive UVB-induced actions, e.g., secretion of β-endorphin Synthesis of many vitamin D compounds with unknown physiologic relevance	-
